# Peroxide of Hydrogen

**Published:** 1885-04

**Authors:** 


### Peroxide of Hydrogen.

Dr. S. S. Wallian, of Bloomingdale, read a paper on this subject. The leading physical and chemical properties of the substance were mentioned. It was said that it might take the place of ozone for many purposes, as a germicide, etc., while it was perfectly harmless in the form in which it was used in medicine. It acted by parting with a portion of its oxygen, which, being no doubt endowed with the peculiar activity incident to the nascent state, combined directly with septic substances, and thus put a stop to the putrefactive process. The author then gave a summary of its therapeutical applications, with special reference to its use in the treatment of diphtheria. Cases of carbuncle, sloughing ulcer, and septic infection (one of each) were then alluded to as having occurred in the author's practice and having been treated with the peroxide with brilliant results.

Tooth in the Antrum of Highmore.—A case of considerable interest is chronicled in the Proceedings of the New York Pathological Society. Dr. J. A. Wyeth presented one of the permanent teeth which he had removed from the antrum of Highmore in a woman 26 years of age, who had suffered from difficulty referred to that locality for about thirteen years, dating from an attack of measles or scarlet fever. A dentist had drawn a tooth, from the locality of which pus discharged from time to time for some years afterwards. Dr. Wyeth extracted two of the teeth, and entered the antrum for the purpose of cleaning it and establishing drainage, when he came upon the foreign body in the form of a tooth. It was the only authenticated case of the kind of which he had any knowledge. He asked the members if they had a similar experience.

MRS. LOUISA REED STOWELL, well known in this city, the only lady instructor in the University of Michigan, and the author of several treatises on microscopical subjects, has just been elected a member of the Royal Microscopical Society of London, Eng., being the third lady ever elected.

